# Metabolic predictors of ischemic heart disease and cerebrovascular attack in elderly diabetic individuals: difference in risk by age

**DOI:** 10.1186/1475-2840-12-10

**Published:** 2013-01-09

**Authors:** Toshio Hayashi, Atsushi Araki, Seinosuke Kawashima, Hirohito Sone, Hiroshi Watanabe, Takashi Ohrui, Koutaro Yokote, Minoru Takemoto, Kiyoshi Kubota, Mitsuhiko Noda, Hiroshi Noto, Koichiro Ina, Hideki Nomura

**Affiliations:** 1 Department of Geriatrics, Nagoya University Graduate School of Medicine, 65 Tsuruma-cho, Showa-ku, Nagoya, Japan; 2 Division of Diabetes, Metabolism and Endocrinology, Tokyo Metropolitan Geriatric Hospital, Tokyo, Japan; 3 Osaka Saiseikai Nakatsu Hospital, Osaka, Japan; 4 Department of Internal Medicine, Endocrinology and Metabolism, Niigata Graduate School of Medicine, Niigata, Japan; 5 Department of Clinical Pharmacology and Therapeutics, Hamamatsu University School of Medicine, Hamamatsu, Japan; 6 Department of Geriatric Medicine, Tohoku University School of Medicine, Sendai, Japan; 7 Division of Diabetes, Metabolism and Endocrinology, Department of Internal Medicine, Chiba University Hospital, Chiba, Japan; 8 Department of Pharmacoepidemiology, Faculty of Medicine, University of Tokyo, Tokyo, Japan; 9 Department of Diabetes and Metabolic Medicine, National Center for Global Health and Medicine, Tokyo, Japan; 10 Department of Geriatrics, Nagoya Kita Hospital, Nagoya, Japan

**Keywords:** Elderly, Diabetes mellitus, Cardiovascular diseases, HDL-C, LDL-C/HDL-C ratio

## Abstract

**Background:**

High LDL-cholesterol (LDL-C) and glucose levels are risk factors for ischemic heart disease (IHD) in middle-aged diabetic individuals; however, the risk among the elderly, especially the very elderly, is not well known. The aim of this study was to identify factors that predict IHD and cerebrovascular attack (CVA) in the elderly and to investigate their differences by age.

**Methods:**

We performed a prospective cohort study (Japan Cholesterol and Diabetes Mellitus Study) with 5.5 years of follow-up. A total of 4,014 patients with type 2 diabetes and without previous IHD or CVA (1,936 women; age 67.4 ± 9.5 years, median 70 years; <65 years old, n = 1,261; 65 to 74 years old, n = 1,731; and ≥ 75 years old, n = 1,016) were recruited on a consecutive outpatient basis from 40 hospitals throughout Japan. Lipids, glucose, and other factors related to IHD or CVA risk, such as blood pressure (BP), were investigated using the multivariate Cox hazard model.

**Results:**

One hundred fifty-three cases of IHD and 104 CVAs (7.8 and 5.7/1,000 people per year, respectively) occurred over 5.5 years. Lower HDL-cholesterol (HDL-C) and female gender were correlated with IHD in patients ≥75 years old (hazard ratio (HR):0.629, P < 0.01 and 1.132, P < 0.05, respectively). In contrast, systolic BP (SBP), HbA1C, LDL-C and non-HDL-C were correlated with IHD in subjects <65 years old (P < 0.05), and the LDL-C/HDL-C ratio was correlated with IHD in all subjects. HDL-C was correlated with CVA in patients ≥75 years old (HR: 0.536, P < 0.01). Kaplan-Meier estimator curves showed that IHD occurred more frequently in patients <65 years old in the highest quartile of the LDL-C/HDL-C ratio. In patients ≥75 years old, IHD and CVA were both the most frequent among those with the lowest HDL-C levels.

**Conclusions:**

IHD and CVA in late elderly diabetic patients were predicted by HDL-C. LDL-C, HbA1C, SBP and non-HDL-C are risk factors for IHD in the non-elderly. The LDL-C/HDL-C ratio may represent the effects of both LDL-C and HDL-C. These age-dependent differences in risk are important for developing individualized strategies to prevent atherosclerotic disease.

**Trial registration:**

UMIN-CTR, UMIN00000516

## Introduction

Type 2 diabetes mellitus, dyslipidemia and aging are independent risk factors for cardiovascular diseases, such as ischemic heart disease (IHD). Within diabetic individuals, lipids, especially LDL-cholesterol (LDL-C), blood pressure (BP), and diabetic control are risk factors for IHD [1-4]. For example, the United Kingdom Prospective Diabetes Study (UKPDS) showed the importance of BP, lipids, and diabetic control in the prevention of IHD in newly diagnosed diabetic individuals(mean age 53 years, range 25–65 years), and subsequent studies have confirmed these findings [1,2]. However, the risk factors for IHD or cerebrovascular attack (CVA) in elderly diabetic individuals (older than 65 years), particularly in late elderly diabetic individuals (older than 75 years), have not been identified.

In Western countries, the evidence suggests that middle-aged diabetic individuals have an IHD risk similar to that of non-diabetic patients who have experienced a myocardial infarction, and the guidelines for diabetes treatment recommend that the LDL-C level should be less than 100 mg/dl, which is similar to the recommendation for the secondary prevention of myocardial infarction [5,6]. However, it is unknown whether the same risk exists for elderly diabetic individuals. Additionally, many guidelines recommend strict control of LDL-C levels to prevent atherothrombotic diseases, especially in diabetic patients, yet recommend the same HDL-cholesterol (HDL-C; 40 mg/dl) and triglyceride (TG; 150 mg/dl) levels as for non-diabetic individuals [5-7]. There are few reports on the absolute risk conferred by HDL-C and TG in elderly diabetic patients.

Additionally, diabetes can either develop in the elderly or continue through old age after an earlier onset. Even in elderly individuals without diabetes, postprandial hyperglycemia occurs because of a delay in insulin secretion in response to feeding and may contribute to an increase in the number of elderly diabetic patients [8]. The International Diabetes Federation (IDF) reports that the number of diabetic patients increased from 30 million in 1987 to 246 million in 2007 (7% of adults) and speculates that it will increase to 380 million by 2027 [9]. In Japan, 30% of diabetic individuals were elderly in 1997 (13% of the elderly suffered from diabetes mellitus), which increased to 40% in 2007 (17% of the elderly). Furthermore, individuals older than 75 (13 million) comprise over 10% of the total population. However, no large-scale investigations have focused on type 2 diabetes mellitus in the elderly, especially in the late elderly, or those older than 75 [10]. Thus, evaluating the metabolic predictors of atherosclerotic diseases, such as IHD and CVA, in elderly diabetic individuals is important. For these reasons, we organized the Japan Cholesterol and Diabetes Mellitus Study (JCDM) to evaluate which factors can predict IHD or CVA in diabetic patients, including the elderly. Our elderly sample population included 1,016 late elderly, who were older than 75 and performed independent daily life activities at outpatient clinics [11].

## Materials and methods

### Subjects

**Figure 1:**
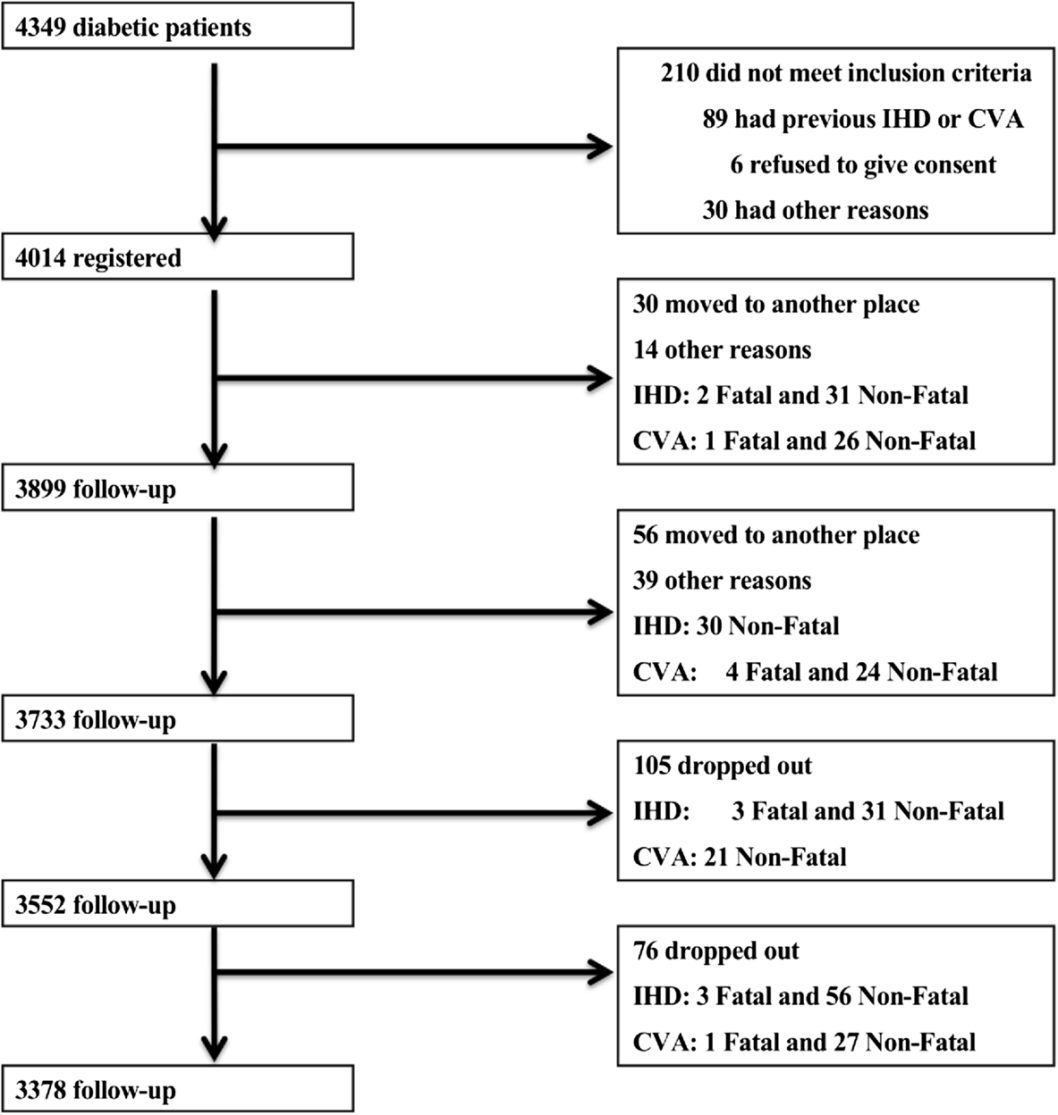
**Study profile.** “Months” indicates months after March 2005.

The JCDM is a prospective, cohort study that consists of 4,014 Japanese diabetic individuals from 40 hospitals throughout Japan who were recruited on a consecutive outpatient basis between September 2004 and March 2005 (1,936 women; mean age 67.4 ± 9.5 years, median age 70 years; Figure 1) [11]. The JCDM protocol, which is in accordance with the provisions of the Declaration of Helsinki, received ethical approval from the institutional review boards of all the participating institutes. Written informed consent was obtained from all patients. The criteria from the American Diabetes Association for type 2 diabetes mellitus diagnosis were used [6]. Patients with previous IHD (myocardial infarction, unstable angina pectoris, angioplasty, or bypass grafting) or CVA (recent stroke with admission within the past 24 months) were excluded, as were patients whose medical records concerning plasma lipids (TG, HDL-C and total cholesterol or LDL-C) were not provided. The other exclusion criteria were a history or complication of serious heart disease (e.g., severe arrhythmia, heart failure, cardiomyopathy, valvular disease, or congenital disease), serious hepatic or renal disease with admission within the past 24 months, malignant disease, intention to undergo surgery, any illness with a poor prognosis of less than one year, and judgment by the physician in charge that the patient was not suitable for the study.

At 24 months (2007), 92.3% of the enrolled patients were followed up, and 84.1% were followed up at 66 months (2010). Patients were divided into groups based on age at registration: younger than 65 (non-elderly, n = 1,267), 65 to 74 years old (early elderly, n = 1,731) and older than 75 (late elderly, n = 1,016). These age categories are used frequently in Japan for the study of elderly patients and for health care insurance purposes [12].

### Outcome measurements

The primary endpoints were the incidence of IHD and CVA, specifically fatal and non-fatal myocardial infarction and other non-fatal events, including unstable angina pectoris, angioplasty, stenting, coronary artery bypass grafting and stroke. Detailed definitions of each event are shown below. Transient ischemic attacks were included only if definite focal lesions from the attack were confirmed by head CT or MRI.

### Risk factor assessment

Metabolic factors, such as the levels of plasma lipids, fasting plasma glucose (FPG), and HbA1C and BP, were measured at enrollment. The serum LDL-C level was calculated using the Friedewald equation, except in the case of a TG level higher than 400 mg/dl, in which case the LDL-C data were recorded as ‘missing.’ Information about previous history of IHD and stroke and findings from a 12-lead ECG were obtained for all patients to assess cardiovascular disease at baseline. The study was approved by the institutional review boards and by the safety monitoring board every year. The organizing committee confirmed all cardiovascular events annually. The guidelines of the Japan Atherosclerosis Society (2002) state that the LDL-C level should be less than 120 mg/dl and that the HDL-C level should be higher than 40 mg/dl in diabetic individuals; these clinical guidelines were likely followed by the physicians who were treating these patients at the time of the study [12].

### Statistical methods

The results are presented as the means ± SD. All statistical analyses were performed using JMP software (SAS Institute, Inc., Cary, NC). The incidences of IHD and CVA were analyzed in relation to the aforementioned risk factors. Cox multivariate regression analyses were used. Because LDL-C/HDL-C interacts strongly with LDL-C and HDL-C and because non-HDL-C interacts with triglyceride and LDL-C, we analyzed non-HDL-C and LDL-C/HDL-C separately. In other words, common factors (gender, age, duration of diabetes, HbA1C, FPG, systolic BP (SBP), and diastolic BP (DBP)),TG, LDL-C and HDL-C were analyzed first. Then, non-HDL-C and common factors were analyzed. Finally, LDL-C/HDL-C, common factors and TG were analyzed. Values of P < 0.05 were considered statistically significant.

**Definition of major events.** Major events such as IHD and CVA were defined as follows.

1. Definite fatal and nonfatal myocardial infarction (1 or more of the following criteria must be met):

a) Diagnostic ECG at the time of the event.

b) Ischemic cardiac pain (and/or unexplained acute left ventricular failure) and diagnostic enzyme levels.

c) Ischemic cardiac pain and/or unexplained acute left ventricular failure with both equivocal enzyme levels and equivocal ECG.

d) Diagnostic enzyme levels and equivocal ECG.

e) Angiographic evidence of major artery occlusion with appropriate ventriculographic wall motion abnormality where a previous angiogram showed no such abnormality.

f) Postmortem examination.

2. Angina pectoris (stable or unstable, both of the following criteria must be met):

a) Ischemic cardiac pain relieved by nitrates.

b) Equivocal ECG.

3. Ischemic stroke (1 of the following conditions must be met):

a) Rapid onset of focal neurologic deficit lasting at least 24 h or leading to death, plus evidence from neuroimaging (computed tomography or magnetic resonance imaging) showing cerebral/cerebellar infarction or no abnormality, or postmortem examination showing cerebral and/or cerebellar infarction.

b) Rapid onset of global neurological deficit (e.g., coma) lasting at least 24 h or leading to death, plus evidence from neuroimaging showing infarction, or postmortem examination showing infarction.

c) Focal neurological deficit (mode of onset uncertain) lasting at least 24 h or leading to death, plus evidence from neuroimaging showing infarction, or postmortem examination showing infarction.

4 Primary intracerebral hemorrhage (1 of the following conditions must be met):

a) Rapid onset of focal neurological deficit lasting at least 24 h or leading to death, plus neuroimaging or postmortem examination showing primary intracerebral and/or cerebellar hemorrhage.

b) Rapid onset of global neurologic deficit (e.g., coma) lasting at least 24 h or leading to death, plus evidence from neuroimaging or postmortem examination showing primary intracerebral and cerebellar hemorrhage.

c) Focal neurologic deficit (mode of onset uncertain) lasting at least 24 h or leading to death, plus evidence from neuroimaging or postmortem examination showing primary intracerebral and/or cerebellar hemorrhage.

In this study, intracerebral hemorrhage was not included in the variable CVA (stroke) because its pathophysiology is reported to be different from other atherosclerotic diseases, such as stroke and ischemic heart disease.

## Results

### Subject characteristics

Table 1 presents the following subject characteristics: plasma lipid levels, including LDL-C, TG, and HDL-C; other relevant metabolic measures, such as HbA1C level, FPG level, and SBP and DBP; the duration of diabetes; and the number of patients who were prescribed medications for hypertension, dyslipidemia, and diabetes, as well as the type, upon enrollment. The levels of HbA1C and HDL-C were not different by age group. Dyslipidemia was observed in 79.1% of patients, and anti-hyperlipidemic drugs were prescribed for 57.3% of the total population, of which 83% were HMG-CoA reductase inhibitors (statins). Statins and insulin were prescribed with the same frequency for late elderly patients as for non-elderly patients. Insulin and oral agents for diabetes treatment were prescribed for 23.9% and 70.5% of the late elderly and non-elderly individuals, respectively. Agents for hypertension and diabetes were prescribed more often in late elderly patients than in non-elderly patients. There were also significant differences in several other factors among the age groups.

**Table 1:** Basic patient profile

**n = 4014**	**Total**		**<65 years**		**65-74 years**		**≥75 years**		**P1**	**Male**		**Female**		**P2**
	**n = 4014**		**n = 1267**		**n = 1731**		**n = 1016**			**n = 2078**		**n = 1936**		
	**Mean**	**SD**	**Mean**	**SD**	**Mean**	**SD**	**Mean**	**SD**		**Mean**	**SD**	**Mean**	**SD**	
**Gender (% male)**	**51.2**	**53.1**	**49.8**	**49.9**	*	**100.0**	**0**	**-**						
**Age (yrs,mean/median)**	**67.9/70**	**2.0**	**56.5/58**	**7.0**	**70.0/70**	**2.7**	**78.8/78**	**3.5**	**-**	**67.0/69**	**10.0**	**69.7/70**	**8.7**	**
**Duration of DM (months)**	**177.9**	**70.0**	**156.3**	**64.5**	**186.1**	**101.2**	**190.7**	**104.3**	***	**191.6**	**108.9**	**169.8**	**79.7**	**
**HbA1C (%)**	**7.70**	**0.80**	**7.74**	**1.38**	**7.72**	**1.18**	**7.62**	**1.14**	**0.17**	**7.61**	**1.25**	**7.79**	**1.20**	**
**FPG (mg/dl)**	**149.8**	**30.3**	**157.9**	**53.2**	**146.4**	**45.7**	**145.4**	**42.0**	**	**150.9**	**48.8**	**146.9**	**44.9**	**0.17**
**SBP (mmHg)**	**137.3**	**11.7**	**133.1**	**17.3**	**135.3**	**16.8**	**146.0**	**17.5**	**	**133.8**	**17.2**	**135.9**	**17.1**	*
**DBP (mmHg)**	**74.0**	**7.3**	**76.1**	**11.8**	**73.5**	**10.6**	**72.3**	**10.7**	***	**74.5**	**11.1**	**73.3**	**10.9**	*
**TG (mg/dl)**	**138.2**	**53.8**	**159.4**	**156.3**	**128.5**	**82.0**	**128.4**	**73.0**	***	**142.9**	**126.3**	**131.3**	**83.7**	**0.18**
**LDL-C (mg/dl)**	**118.2**	**21.3**	**121.6**	**33.8**	**117.6**	**32.1**	**115.0**	**29.3**	*	**115.0**	**31.8**	**121.2**	**31.4**	**
**HDL-C (mg/dl)**	**55.8**	**10.7**	**54.8**	**15.6**	**55.4**	**15.5**	**57.7**	**15.8**	**0.38**	**53.42**	**15.4**	**56.78**	**15.7**	**
**Non-HDL-C (mg/dl)**	**145.8**	**23.4**	**153.2**	**40.3**	**143.3**	**35.5**	**141.0**	**31.8**	*	**143.6**	**36.9**	**147.5**	**35.6**	*
**LDL-C/HDL-C**	**2.31**	**0.74**	**2.41**	**1.17**	**2.31**	**1.21**	**2.27**	**0.88**	*	**2.33**	**0.95**	**2.33**	**1.28**	**0.21**
**Agents for HT (%)**	**55.5**		**49.3**		**56.0**		**62.3**		**	**48.5**		**62.0**		***
**ACEI/ARB**	**39.7**		**36.9**		**40.4**		**41.9**		**0.65**	**35.8**		**43.4**		*
**CCB**	**41.2**		**32.4**		**43.7**		**52.6**		**0.31**	**36.0**		**46.5**		*
**Others**	**28.3**		**22.5**		**31.8**		**31.4**		**0.69**	**28.9**		**26.7**		**0.66**
**Agents for DL (%)**	**57.3**		**63.8**		**54.8**		**52.6**		**	**52.1**		**60.1**		***
**Strong statins**	**29.6**		**31.1**		**28.3**		**25.4**		**0.36**	**29.7**		**29.5**		**0.89**
**Classical statins**	**53.3**		**47.0**		**58.0**		**61.6**		**0.11**	**51.9**		**55.0**		**0.23**
**Fibrates**	**8.9**		**12.0**		**6.5**		**6.8**		**0.13**	**9.8**		**7.9**		**0.18**
**Others**	**8.2**		**9.9**		**7.2**		**6.2**		**0.10**		**8.6**		**7.6**		**0.22**
**Agents for DM (%)**	**86.6**		**76.9**		**91.6**		**90.3**		**	**85.1**		**88.6**		*	
**Insulin**	**23.9**		**24.4**		**24.6**		**21.7**		**0.42**	**28.0**		**32.4**		*	
**Sulfonylurea**	**49.5**		**41.5**		**51.3**		**53.7**		**0.21**	**50.0**		**48.9**		**0.29**	
**Others**	**26.1**		**34.6**		**21.4**		**24.5**		**0.19**	**26.0**		**22.0**		*	
**IHD (/1000 year)**	**9.68**		**8.84**		**10.04**		**9.87**		**0.97**	**10.26**		**9.47**		**0.32**	
**CVA (/1000 year)**	**6.78**		**4.45**		**7.44**		**7.56**		**0.21**	**7.02**		**5.72**		**0.27**	

P1: Differences in each factor among ages. P2: Differences in each factor between genders. HbA1C:NGSP, *P < 0.05, **P < 0.01, ***P < 0.001.

### IHD and CVA incidence

One hundred fifty-three cases of IHD and 104 CVAs occurred during the 5.5 years of the study, which represented incidences of 7.9 and 5.6 per 1,000 patients per year, respectively. The number of deaths was 59 (3.1/1,000 patient-years) over the 5.5 years (Table 2, Figure 1).

**Table 2:** Risk factors for IHD and CVA by Cox multivariate models in each age group (IHD, upper; CVA, lower)

**n = 4014**	**Total (n = 4014)**			**<65 years (n = 1267)**			**65-74 years (n = 1731)**			**≥75 years (n = 1016)**		
**IHD**	**Adjusted HR**	**95% CI**	**P**	**Adjusted HR**	**95% CI**	**P**	**Adjusted HR**	**95% CI**	**P**	**Adjusted HR**	**95% CI**	**P**
**Gender (women vs. men)**	1.103	0.972–1.268	0.197	1.044	0.967–1.073	0.456	1.085	0.978–1.210	0.101	**1.132**	**0.992–1.278**	0.019*
**Age (per 10 years)**	1.013	0.972–1.066	0.328	1.022	0.977–1.079	0.229	**1.054**	**1.002–1.106**	0.049*	1.005	0.871–1.139	0.682
**Duration of Diabetes (months)**	0.995	0.988–1.003	0.053	1.001	0.991–1.008	0.582	**0.993**	**0.985–0.999**	0.033*	**0.992**	**0.982–0.999**	0.023*
**HbA1C (per 1**%**)**	**1.171**	**1.001–1.356**	0.047*	**1.327**	**1.025–1.686**	0.032*	1.219	0.973–1.487	0.083	0.792	0.479–1.059	0.134
FPG (per 10 mg/dl)	1.004	0.997–1.008	0.432	1.005	0.996–1.013	0.355	1.004	0.997–1.009	0.592	0.999	0.987–1.007	0.761
**SBP(per 10 mmHg)**	1.008	0.995–1.021	0.186	**1.030**	**1.000–1.055**	0.035*	1.014	0.994–1.037	0.175	0.986	0.954–1.014	0.331
DBP(per 10 mmHg)	0.995	0.978–1.015	0.618	0.982	0.948–1.024	0.386	0.980	0.950–1.011	0.206	1.027	0.986–1.073	0.202
TG (quartile)	1.005	0.889–1.166	0.555	1.002	0.996–1.006	0.502	1.108	0.997–1.220	0.065	1.001	0.961–1.046	0.454
**LDL-C** (quartile)	**1.318**	**1.103–1.585**	0.023*	**1.571**	**1.128–2.524**	0.016*	1.050	0.932–1.176	0.112	1.156	0.998–1.309	0.054
**HDL-C** (quartile)	**0.751**	**0.611–0.917**	0.005**	0.828	0.646–1.017	0.072	0.987	0.966–1.008	0.204	**0.629**	**0.401–0.856**	0.001**
Non-HDL-C (quartile)	1.023	0.981–1.072	0.075	**1.025**	**1.001–1.121**	0.044*	1.073	0.982–1.161	0.086	0.941	0.791–1.102	0.621
**LDL-C/HDL-C** (quartile)	**1.583**	**1.298–1.945**	0.001**	**2.324**	**1.516–3.795**	0.001**	**1.359**	**1.028–1.824**	0.021*	**1.407**	**1.015–2.592**	0.029*
**CVA**	**Adjusted HR**	**95% CI**	**P**	**Adjusted HR**	**95% CI**	**P**	**Adjusted HR**	**95% CI**	**P**	**Adjusted HR**	**95% CI**	**P**
Gender	1.164	0.985–1.296	0.351	1.014	0.897–1.240	0.655	1.208	0.896–1.526	0.112	0.953	0.912–1.012	0.063
**Age**	1.015	0.986–1.039	0.282	1.002	0.957–1.076	0.754	1.007	0.916–1.166	0.537	**1.103**	**1.002–1.217**	0.048*
Duration of Diabetes	0.998	0.992–1.001	0.206	1.003	0.987–1.017	0.709	0.996	0.989–1.001	0.096	0.999	0.991–1.005	0.818
HbA1C	1.001	0.790–1.214	0.128	1.019	0.691–1.401	0.814	0.997	0.855–1.222	0.569	0.928	0.822–1.010	0.059
FPG	1.005	0.995–1.005	0.803	1.003	0.990–1.018	0.741	1.002	0.995–1.008	0.592	0.998	0.986–1.008	0.711
SBP	1.009	0.993–1.024	0.276	1.024	0.988–1.055	0.185	1.015	0.992–1.037	0.206	0.989	0.957–1.018	0.458
DBP	0.998	0.978–1.020	0.846	0.995	0.958–1.046	0.831	0.981	0.948–1.016	0.278	1.024	0.978–1.074	0.317
TG	1.132	0.908–1.302	0.156	1.053	0.658–1.742	0.833	1.253	0.900–1.780	0.184	1.169	0.746–1.853	0.497
**LDL-C**	1.009	0.912–1.191	0.675	**1.005**	**1.001–1.100**	0.047*	1.015	0.892–1.136	0.714	0.997	0.982–1.012	0.631
**HDL-C**	**0.742**	**0.596–0.901**	0.003**	0.715	0.591–1.191	0.200	**0.750**	**0.494–1.000**	0.049*	**0.536**	**0.320–0.851**	0.007**
**Non-HDL-C**	0.981	0.945–1.019	0.206	**1.021**	**1.003–1.141**	0.045*	0.942	0.872–1.013	0.172	1.012	0.954–1.077	0.226
LDL-C/HDL-C	1.180	0.951–1.477	0.132	1.271	0.819–2.232	0.263	1.114	0.853–1.582	0.356	1.209	0.803–1.847	0.364

**The top panels show the analyses of IHD for subjects aged <65 years (left), 65–74 years (middle) and ≥75 years (right). The lower panels show the incidence of CVA. Bold indicate statistically significant factors. Hazard ratios and 95% CIs are shown. The ratio of males to females was 1. As LDL-C/HDL-C interacts strongly with LDL-C and HDL-C, and non-HDL-C interacts triglyceride and LDL-C, analysis of non-HDL-C and LDL-C/HDL-C were separately shown in methods section.**

**Figure 2:**
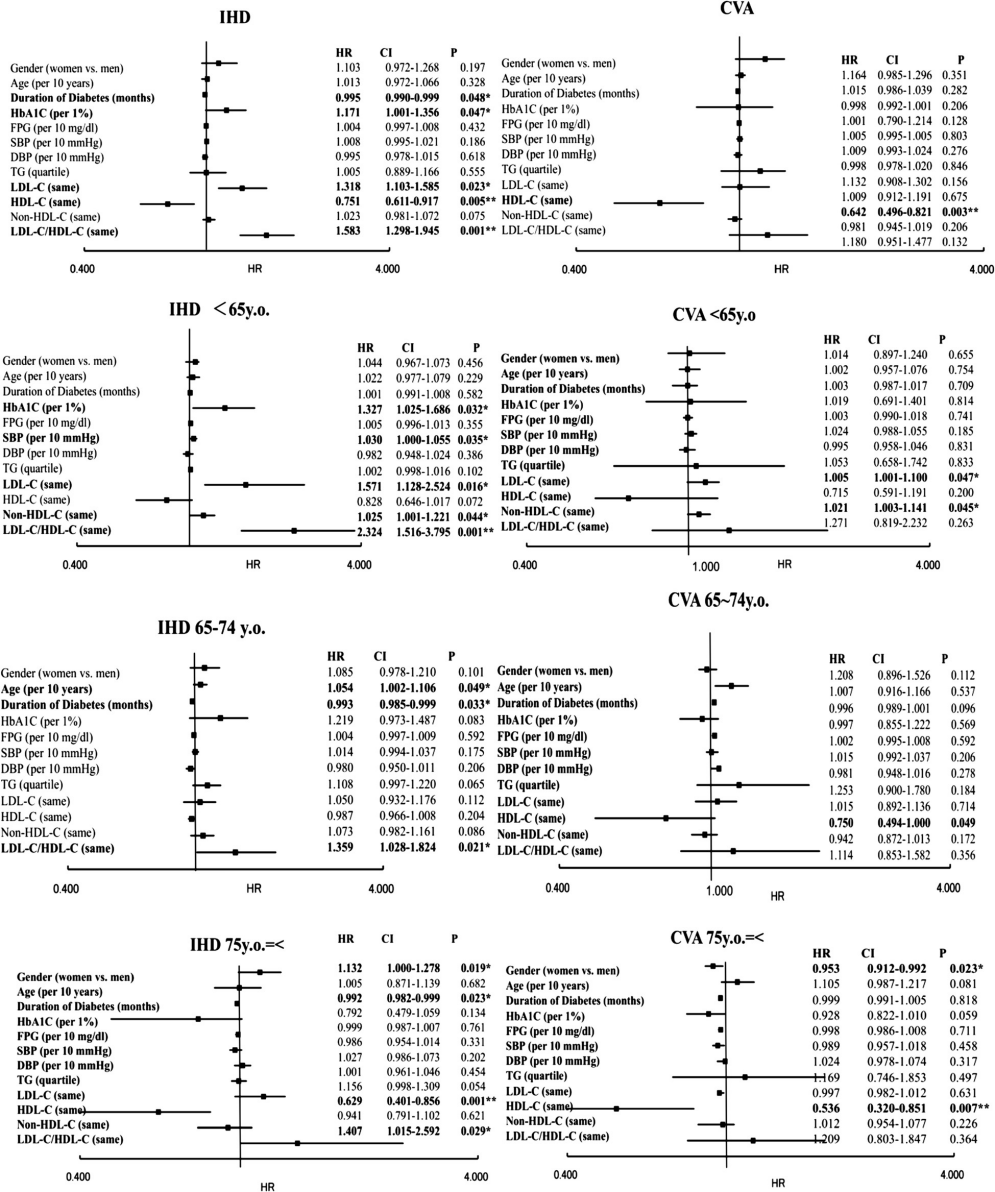
**Risk factors for IHD and CVA by Cox multivariate models in representative age groups (IHD, Left; CVA, Right).** The upper panels show the analyses of IHD for those younger than 65 years old (Left) and CVA for those younger than 65 years old (Right). The lower panels show the analyses of IHD (Left) and CVA (Right) for those equal to or older than 75 years. HR stands for Hazard Ratio (vertical bar shows 1). Bold characters indicate statistically significant factors. The right side of each figure shows Hazard Ratios and 95% CIs. Because LDL-C/HDL-C interacts strongly with LDL-C and HDL-C and because non-HDL-C interacts with triglyceride and LDL-C, we analyzed non-HDL-C and LDL-C/HDL-C separately. In other words, common factors (gender, age, duration of diabetes, HbA1C, FPG, systolic BP (SBP), and diastolic BP (DBP)), TG, LDL-C and HDL-C were analyzed. Then, non-HDL-C and common factors were analyzed. Finally, LDL-C/HDL-C, common factors and TG were analyzed.

The relationships between IHD or CVA and the background factors, such as LDL-C level, in each age group were analyzed by Cox proportional regression analyses (Table 2, Figure 2).

As described in the methods, non-HDL-C and LDL-C/HDL-C were analyzed separately from other lipids, such as LDL-C and triglyceride or HDL-C. However, significant factors were the same in total and in each generation group, although the HR and CI of common factors (gender, age, duration of diabetes, HbA1C, FPG, systolic BP (SBP), and diastolic BP (DBP)) were slightly different in each (data not shown for the HR and CI of common factors in the analyses of non-HDL-C and LDL-C/HDL-C).

**Figure 3:**
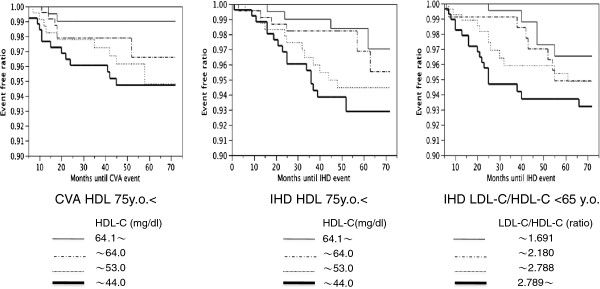
**The relationships of HDL-C levels and the LDL-C/HDL-C ratio with IHD and CVA in quartile categories for each age group based on Kaplan-Meier estimator curves. Left**: The LDL-C/HDL-C ratio correlates with IHD. The figure shows the data for individuals aged <65 years. **Center:** The HDL-C level was inversely correlated with IHD, particularly in individuals aged >75 years. **Right:** The association between CVA and HDL-C was more prominent in those individuals aged ≥ 75 years. Quartile categories HDL-C: <44.0, 44.1-53.0, 53.1-64.0, >64.1 mg/dl LDL-C/HDL-C: <1.691,1.692-2.180, 2.181-2.787, >2.789.

In the total patient population, the levels of HbA1C, LDL-C, and HDL-C, and the LDL-C/HDL-C ratio were significantly related to IHD, and only the HDL-C level was significantly related to a CVA. The HbA1C level, SBP, and LDL-C levels were significantly correlated with IHD in patients less than 65 years old, while the variables female gender, short duration of diabetes and HDL-C level were correlated with IHD in patients older than 75. Because the non-HDL-C level and the LDL-C/HDL-C ratio have been proposed as markers representing all types of lipids, we included them in a separate model (excluding LDL-C, triglyceride and HDL-C levels to avoid the interactive effect on non-HDL-C, or excluding LDL-C and HDL-C levels for the LDL-C/HDL-C ratio). The non-HDL-C level was only correlated with IHD in patients younger than 65. The LDL-C/HDL-C ratio was significantly correlated with IHD in patients of all generations. Age and lower HDL levels were correlated with CVA in patients over 75 years old (Table 2, Figure 2). Subsequently, we evaluated the relationships with IHD and CVA according to the quartile categories for each age group by Kaplan-Meier estimator curves. The HDL-C level was inversely correlated with IHD and CVA, particularly in individuals over 75 (Figure 3). The LDL-C/HDL-C ratio tended to correlate with IHD in all individuals (Figure 3). For the variable current smokers, 6.8% of the total population of subjects smoked. By age category, 9.9, 6.7 and 3.8% of patients younger than 65, patients between 65 and 74, and patients older than 75 smoked, respectively. As the duration of diabetes is pretty long, number of present smokers is not many.

## Discussion

### Background and discussion points of the study

The numbers of diabetic elderly and their associated net medical costs have drastically increased in recent decades. The mean life expectancy is now approximately an additional 12 and 16 years at age 75 for males and females in Japan, respectively, although the average life span is 78.9 and 85.6 years, respectively. Consequently, the number of late elderly (individuals older than 75) exceeds 13 million, or 10% of the total Japanese population. Diabetes can either develop in the elderly or continue through old age after an earlier onset, and the numbers of diabetic elderly are increasing. In Japan, 55% of diabetic individuals were elderly in 2007, and approximately 25% were late elderly. These trends are spreading across the world, mainly in developed countries; however, the risk factors for IHD or CVA in late elderly diabetic individuals have not been identified. In the late elderly, atherosclerotic diseases, such as IHD and CVA, are a more frequent cause of death than malignancy. In Canada, diabetic patients are reported to suffer myocardial infarction approximately 14 years earlier than patients without diabetes [13]. However, there is little evidence on the risk and preventive factors for IHD or CVA in the diabetic elderly, and there are no reports on the late elderly [14,15].

Therefore, we organized this study as one of the largest attempts to examine IHD and CVA in middle-aged to elderly diabetic individuals. We defined the age categories as follows: 1) non-elderly: younger than 65, 2) early elderly: from 65 to 74, and 3) late elderly: equal to or older than 75. Sixty-five is usually defined as the threshold for being elderly worldwide [13,16], and 75 is the beginning of the late elderly age in Japan, as defined by health insurance and care insurance systems and the Japan Geriatric Society [12].

### The effect of age on IHD and CVA risk factors

One hundred fifty-three cases of IHD and 104 CVAs occurred, which represents 7.8 and 5.7/1,000 people per year, respectively, over this 5.5-year study, although we defined stroke strictly and excluded cerebral and subarachnoid hemorrhages from this definition. IHD occurs 2 to 3 times more frequently in diabetic individuals compared to the normal Japanese population, and CVA also occurs more frequently in diabetic individuals [17]. The prevalence of IHD and CVA is slightly higher than reported in previous Japanese diabetic studies because we targeted relatively older diabetic individuals [16,17]. However, even in diabetic individuals, the combined frequency of IHD and stroke was slightly lower in the Japanese population than among Caucasians [18].

To look for the candidate metabolic markers that may predict IHD and CVA in various age groups, Cox regression analyses were performed. The analyses showed that higher HbA1C and LDL-C levels, SBP and non-HDL-C were significantly correlated with the occurrence of IHD in subjects <65 years old, which is similar to previous reports [14-16]. The ratio of males/females was not significantly different between patients < 65, patients between 65 and 74, and patients ≥75. A relation between diabetes and ischemic stroke was reported. Patients (59.8 ± 7.2 y.o.) having a history of coronary heart disease with diabetes mellitus exhibited a 2.29-fold increased risk for stroke or TIA during the 4.8- to 8.1-year follow-up period than patients without diabetes. Impaired fasting glucose and hypertension were predictors, while HDL-C was not. These results are fairly consistent with those of the younger patients group (< 65 y.o.) in the present study [19].

In patients ≥75 y.o., a lower HDL-C level was correlated with IHD and CVA. This is a novel finding of the present study. Few data are available on the relationship between elderly type 2 diabetic patients and CVA, particularly among the late elderly [16-18,20]; therefore, the finding of the importance of HDL-C in CVA in the late diabetic elderly may be important. The Kaplan-Meier estimator curves, which are shown in Figure 1, support these findings.

Thus, a lower HDL-C level is an important risk factor for both IHD and CVA among the late elderly diabetic patients in this study. Although the protective effects of higher HDL-C on IHD in the non-elderly are known, the effects on IHD among late elderly diabetics are not known [21]. The CVA and IHD incidences in the late elderly may decrease to the levels found in middle-aged cohorts if higher HDL-C has protective effects on late elderly diabetic individuals and if their levels are easily increased. There are few agents available to increase HDL-C levels, except exercise, and adequate exercise or bodily movement may be necessary even in the elderly. The low HDL-C level may be related to low levels of physical activity in the elderly, which could influence a CVA in many ways that are separate from the HDL-C level. Atherosclerosis is an inflammatory disorder, and HDL-C may preserve endothelial function by increasing endothelial NO [22].

For LDL-C, three large-scale clinical studies on dyslipidemia, which included participants who were up to 75 or 80 years of age, are available [23-25]. Although these studies reported that the reduction in LDL-C by statins decreases IHD (including in diabetic people), the effects were weak in the elderly compared with those in the non-elderly (e.g., Prosper reported that pravastatin, a water-soluble statin, induced a 16% decrease in IHD without any effect on CVA in elderly patients compared to a 21% decrease in non-elderly patients). These data suggest that simply controlling LDL-C may not prevent IHD or CVA in the elderly. There are also no large observational studies on the diabetic elderly older than 75 [26,27]. For example, the international FIELD study analyzed approximately 10,000 patients up to the age of 75 years, with a mean age 63 years [26], and the Swedish NDR-study analyzed 18,673 patients up to 70 years old, with a mean age of 60 years [27]. These large observational studies, analyzing all patients, found LDL-C, non-HDL-C, HDL-C, triglycerides and ratios of LDL-C/HDL-C and total-cholesterol/HDL-C to be significant risk factors for IHD. These data are consistent with our data on participants younger than 65, although those observational studies did not include patients older than 75. To lower LDL-C levels, 57% of the patients in our study had already been prescribed anti-dyslipidemic agents, of which 83% were statins. The average LDL-C level was 120 mg/dl, which matches the guidelines of the Japan atherosclerosis society but not that of the American Heart Association or IDF (100 mg/dl). Although doses and types of anti-dyslipidemic agents were changed often during the study, their effects other than LDL reduction (pleiotropic effects) cannot be evaluated yet.

Our study shows the importance of the LDL-C/HDL-C ratio as well as HDL-C and LDL-C levels, although the strength of these effects is different based on age. The LDL-C/HDL-C ratio was associated with IHD, which may represent the effect of LDL-C levels in the non-elderly and HDL-C levels in the elderly [28]. The non-HDL-C level and the total cholesterol/HDL-C ratio are also proposed markers of atherosclerotic diseases [29,30]. The non-HDL-C level was associated with IHD only among those younger than 65, and the total cholesterol/HDL-C ratio was not significantly associated with IHD (data not shown). We believe that these data are consistent with previous data from non-elderly diabetic individuals because the non-HDL-C level is a reflection of the effect of triglyceride levels, and hyper-triglyceridemia, complicated with metabolic syndrome, occurs more often in non-elderly than in elderly people.

Emerging Risk Factors Collaboration analysis showed the association of non-HDL-C with IHD and CVA. However, in this study, it was associated with CVA only in those younger than 65. The two studies are different in that 1) our cohort consisted only of diabetic patients; 2) in the Collaboration analysis, the mean age was 56.6 y.o., compared to 67.4 y.o. in our study; and 3) in the Collaboration analysis, almost all of the patients were North American or European, whereas our study was Japanese patients only. In the elderly, triglycerides are usually lower than in younger individuals, and non-HDL-C represents triglyceride.

A 1-mg/dl change in HDL-C and/or a 2-mg/dl change in LDL-C reflect a 2% change in the risk for atherosclerotic diseases, and this may be partially consistent within our diabetic elderly study [31]. The LDL-C/HDL-C ratio may reflect the direct effects of both LDL-C and HDL-C levels, which may affect or interact with the progression of atherosclerosis and thrombosis formation more than other lipids, such as chylomicrons and chylomicron remnants, which are represented by the non-HDL-C level or the TC/HDL-C ratio. The fact that elderly individuals have different risk factors than younger individuals could be associated with genetic protection from such events or an accumulation of personal habits that may provide the elderly with protection. For example, differences in single nucleotide polymorphisms (SNP) may be related to the severity of atherosclerosis and, subsequently, to the different effects of predictors by age and should be evaluated in the future [32].

Interestingly, impaired fasting glucose and hypertension were the strongest predictors of risk for ischemic stroke or TIA in metabolic syndrome, and HbA1c had positive associations with glycemia, TG, HDL-C, and TG/HDL-C but not LDL-C in the study of 118 older adults aged 65–95 years, of whom less than 6.5% had an HbA1c of 93% [19,33]. These data is consistent with our data in diabetic patients younger than 65 [33]. Another study evaluated the predictors of stroke stratified by age (at symptom onset: young; <50 years, older; 51–75 years, and oldest; 75 < years) using data collected over a 4-year period from 3,053 subjects with stroke. The metabolic syndrome was the only predictor among the older patients (OR 1.58) but not in the others. Although most patients were not diabetic, these types of studies should be accumulated to evaluate the effect of age on atherosclerotic diseases [34].

## Conclusions

HbA1C, LDL-C, SBP and non-HDL-C in non-elderly diabetic individuals, HDL-C in late elderly diabetic individuals and the LDL-C/HDL-C ratio in all diabetic individuals were associated with IHD in this population. HDL-C was also associated with CVA in late elderly diabetic individuals. The differences in atherosclerotic risk by age must be considered in developing individualized strategies for the prevention of atherosclerotic diseases. Because this was an observational study, we could not analyze the detailed effects of treatment, such as the effect of statins on the risk of IHD or CVA. Although this study targets Japanese, these new findings on metabolic markers in the late elderly could provide additional data for the annotation of cardiovascular risk factors in the diabetic elderly across the world.

## Abbreviations

CVA: Cerebrovascular attack; IHD: Ischemic heart disease; LDL-C: LDL-cholesterol; HDL-C: HDL-cholesterol; TG: Triglyceride; FPG: Fasting plasma glucose; HbA1C: Hemoglobin A1C; SBP: Systolic blood pressure; UKPDS: United Kingdom Prospective Diabetes Study.

## 

All authors take responsibility for all aspects of the reliability and freedom from bias of the data presented and their discussed interpretation.

## Competing interests

The authors declare that they have no competing interests.

## Authors’ contributions

TH and HN wrote the manuscript and researched the data. AA, SK, HS, HW, TO, KY, MT, KK, MN, HN, and KI contributed to the research and reviewed the manuscript. All authors read and approved the final manuscript.
